# Hydrogel Extinguishants

**DOI:** 10.3390/nano14131128

**Published:** 2024-06-30

**Authors:** Guineng Li, Qiaobo Wang, Guiqun Liu, Mutian Yao, Yue Wang, Yeying Li, Kaiwen Lin, Ximei Liu

**Affiliations:** 1 Jiangxi Province Key Laboratory of Flexible Electronics, Flexible Electronics Innovation Institute, Jiangxi Science and Technology Normal University, Nanchang 330013, China; liguineng2024@163.com (G.L.); wqbglhf2001@163.com (Q.W.); ymt1845638718@163.com (M.Y.); wangyue20240610@163.com (Y.W.); lee_yy01@163.com (Y.L.); 2 School of Materials Science and Technology, North Minzu University, Yinchuan 750021, China; gqliu10b@alum.imr.ac.cn; 3 Department of Materials and Food, University of Electronic Science and Technology of China Zhongshan Institute, Zhongshan 528402, China

**Keywords:** fire extinguishing, hydrogel, fire prevention

## Abstract

The exploitation of clean and efficient fire extinguishing materials has substantial implications for improving disaster prevention, mitigation, and relief capabilities, maintaining public safety, and protecting people’s lives and property as well as the natural environment. Natural polymer hydrogel with high water containment, excellent film formation, high heat insulation, ecofriendliness, and degradability has huge potential in achieving new breakthroughs for developing clean and efficient fire extinguishing materials and products. In recent years, the exploitation of hydrogel extinguishing materials and the fabrication of products has attracted great attention, gradually replacing traditional fire extinguishing products. In this perspective, an in-depth review of the evolution of hydrogels applied for fire extinguishing and prevention is presented. Firstly, the extinguishing principles of hydrogel extinguishants are explained. Secondly, the preparation strategies and evaluation system of the hydrogel extinguishants are emphatically discussed. Although great progress has been made in developing high-performance hydrogel extinguishants, it remains challenging to develop cost-effective, degradable, and easy-to-use hydrogel extinguishants. Additionally, we highlight the importance of considering the commercial aspects of hydrogel extinguishants. Looking into the future, hydrogel extinguishants are promising, but continued investment in research and development is necessary to overcome the challenges.

## 1. Introduction

**Figure 1:**
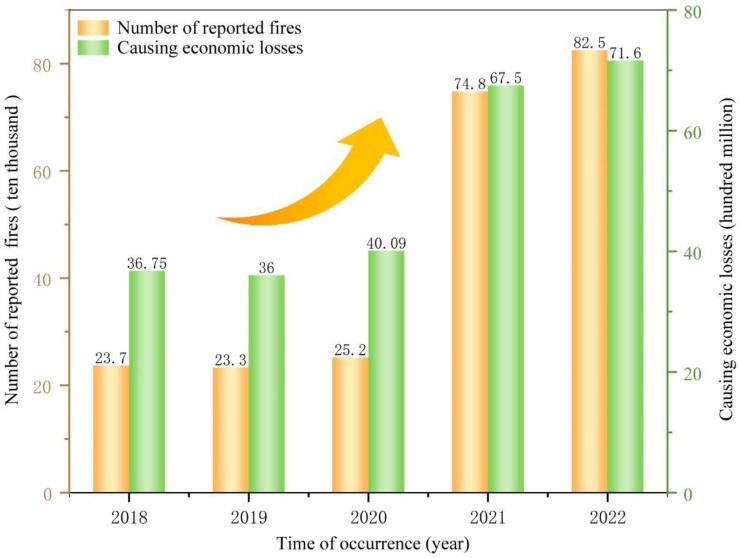
The fire situation in China from 2018 to 2022.

Fires often cause extensive wealth loss and pose a momentous threat to human life while it brings great convenience to people [1,2,3,4,5]. The number of reported fires and the economic losses caused by fires in China are increasing year by year, according to the statistics of the National Fire and Rescue Administration, which reached as high as 825,000, causing economic losses of CNY 7.16 billion by 2022 (Figure 1). According to the latest statistics, a total of 2.138 million fires were reported in 2023. This alarming figure should be taken seriously.

**Figure 2:**
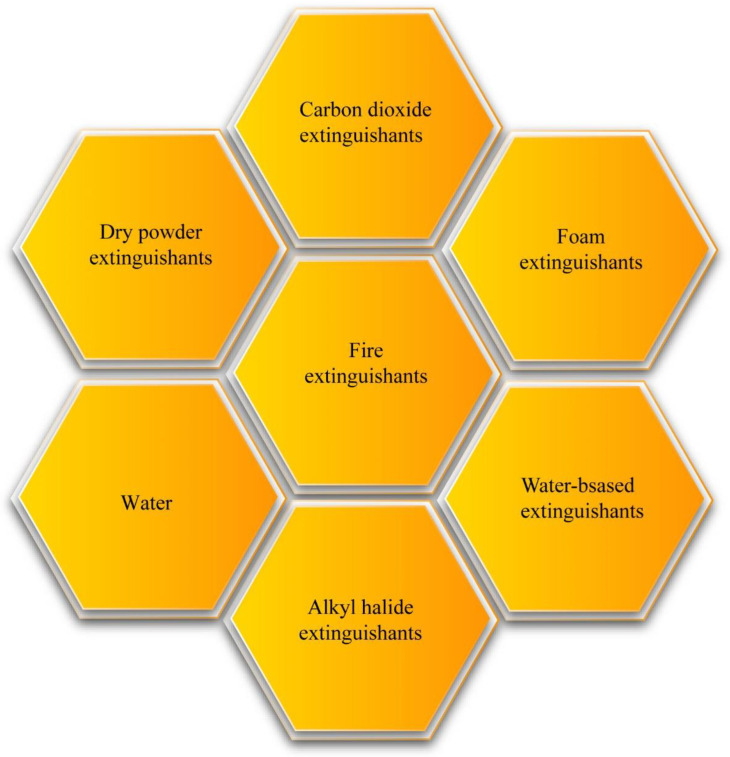
Different types of fire extinguishants.

Fire extinguishants with high-efficiency combustion suppression are essential for preventing fire hazards. Traditional fire extinguishants, such as carbon dioxide, foam, and dry powder, have already fallen short of people’s pursuit of clean and efficient fire extinguishing materials. Recently, new types of water-based extinguishants have attracted significant interest [6,7,8,9,10,11]. Commonly used fire extinguishants, including traditional and new types, are presented in Figure 2. Carbon dioxide fire extinguishants possess the advantages of suppressing the oxygen supply, rapid cooling, and leaving no residue after extinguishing the fire. Another significant feature is that they do not cause harm to objects after use. However, when releasing high concentrations of carbon dioxide, these agents pose serious hazards to personnel, including the risk of asphyxiation and hyperoxia symptoms, necessitating special precautions during use [12]. Foam fire extinguishants, while effectively suppressing smoke and providing lasting thermal insulation, present urgent issues related to corrosion and environmental pollution [13,14,15,16,17]. Dry powder fire extinguishants offer the advantage of both physical and chemical suppression of flame spread. However, the smoke and dust they produce can pose significant hazards to human health and the environment. Similarly, the environmental hazards caused by halogenated alkane fire extinguishants cannot be overlooked [18]. Therefore, there is a growing need for the development of new, clean, and efficient fire extinguishing materials that can overcome the limitations and drawbacks of traditional agents while ensuring safety and environmental sustainability.

**Figure 3:**
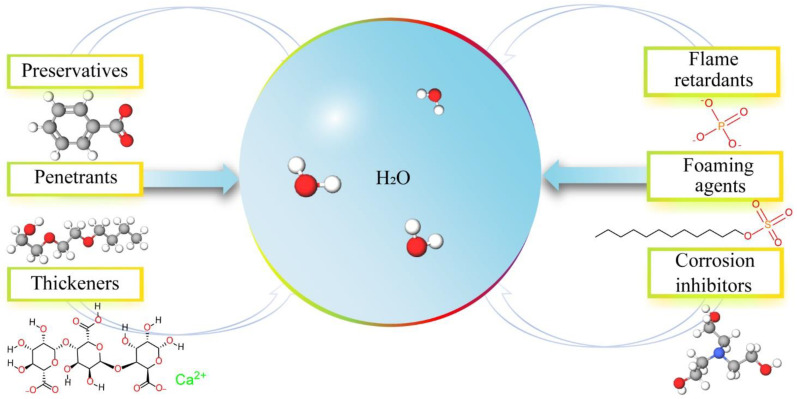
The definition of water-based fire extinguishants.

**Figure 4:**
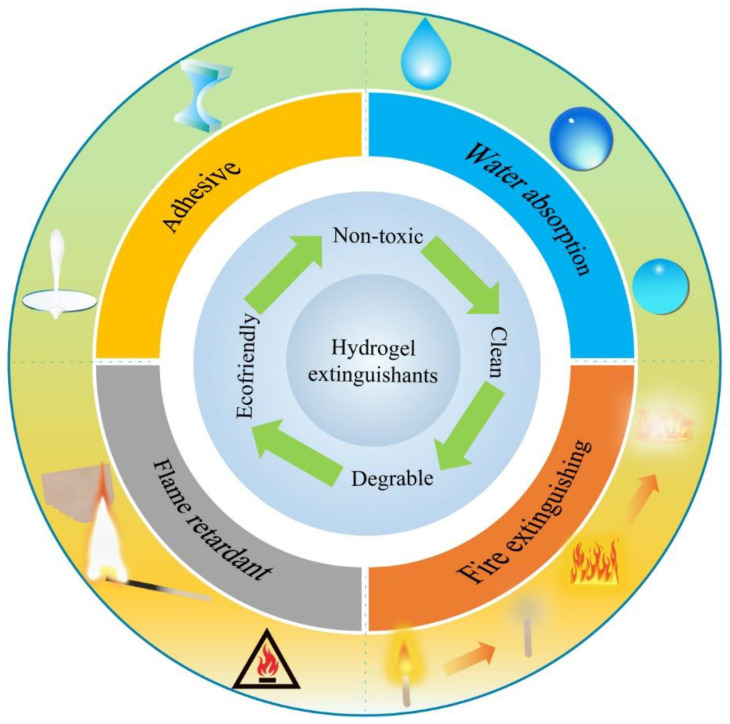
Features and advantages of hydrogel extinguishants.

Water is a naturally non-toxic and highly effective fire extinguishant that can effectively suppress common fire disasters in forests, grasslands, buildings, mines, etc. The combustion matter is cooled to the ignition point or below when a large amount of water is poured, and the evaporated water vapor also plays a role in isolating oxygen. However, a great deal of used water flows and falls from the combustion material side, which contributes to inefficient results [19]. In recent years, water-based extinguishants have gradually attracted widespread attention, and many have moved toward commercial development. As presented in Figure 3, water-based extinguishants are a clean and efficient fire extinguishing agent that enhances the fire extinguishing performance of water by adding various additives such as foaming agents, flame retardants, thickeners, penetrants, etc. Commonly used fire extinguishing additives include sodium lauryl sulfate, ammonium phosphate, calcium alginate, sodium benzoate, triethanolamine, and butyl glycol ether, among others. As one of the water-based extinguishants, hydrogel extinguishants for fire extinguishing and prevention have been proposed due to their excellent water absorption, adhesive [20,21], and flame-retardant properties [22,23,24,25,26,27,28]. Features and advantages of hydrogel extinguishants are summarized in Figure 4.

**Figure 5:**
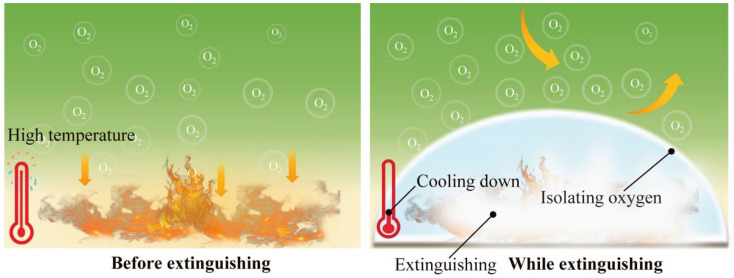
The fire extinguishing principle of hydrogel extinguishants.

Hydrogel extinguishants for firefighting exhibit many superiorities, including water storage, as well as sealing and carrying capacity, compared to pure water [29,30,31,32,33]. Simultaneously, hydrogel extinguishants exhibit better cooling, isolation of oxygen, reduced carbon monoxide production, and other properties than traditional fire extinguishants [34]. Hydrogel extinguishants have been proven to be an ideal material for firefighting, owing to their unique performance, including filling with a large amount of water. Water possesses high molar heat capacity and vaporization enthalpy. Massive water in hydrogel can absorb heat and reduce the temperature to suppress fire [35]. Significantly, hydrogel is non-toxic, clean, degradable, and ecofriendly, making it a promising candidate for commercial promotion. The principles of hydrogel extinguishants are shown in Figure 5.

**Figure 6:**
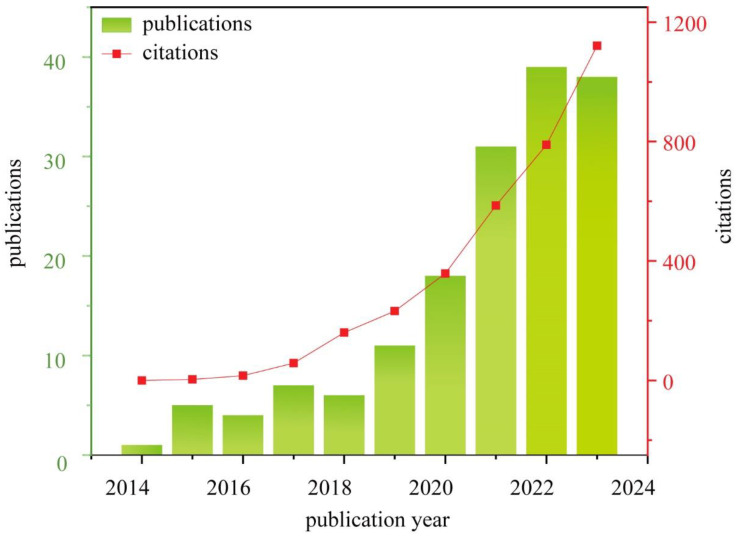
The statistics of publication on hydrogel for fire extinguishing and prevention.

Certainly, there have been many publications on hydrogel research for fire extinguishing and prevention in recent years, and brief statistics are shown in Figure 6. The number of publications has increased rapidly, particularly in the last two years. This phenomenon undoubtedly demonstrates the popularity of hydrogel for fire extinguishing and prevention. However, according to our study, there is no comprehensive review of this topic.

In view of the above-mentioned facts, this paper provides a comprehensive review of the evolution of hydrogel applied for fire extinguishing and prevention, preparation strategies, and evaluation systems of hydrogel extinguishants. We have also discussed the commercial aspects of hydrogel extinguishants as well as the challenges and prospects.

## 2. Evolution of Hydrogel Applied for Fire Extinguishing and Prevention

**Figure 7:**
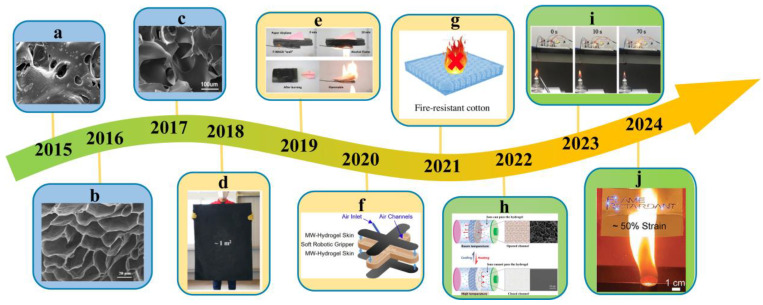
The evolution of hydrogel applied for fire extinguishing and prevention: (**a**) Microstructure of hydrogels. Reprinted with permission from Ref. [39]. Copyright 2015 De Gruyter. (**b**) Side-view SEM images of the anisotropic porous structure of anisotropic graphene aerogels. Reprinted with permission from Ref. [40]. Copyright 2016 Elsevier. (**c**) SEM images of multifunctional polyimide (PI) aerogels. Reprinted with permission from Ref. [32]. Copyright 2017 Elsevier. (**d**) Graphene aerogel bulk. Reprinted with permission from Ref. [41]. Copyright 2018 American Chemical Society. (**e**) Thermal insulation material (F-WAGA) to protect the paper airplane from burning. Reprinted with permission from Ref. [42]. Copyright 2019 John Wiley and Sons. (**f**) MW–hydrogel use in electronic robot skin. Reprinted with permission from Ref. [44]. Copyright 2020 Elsevier. (**g**) New type of three-dimensional network hydrogel that can effectively achieve flame retardancy when layered on fabric. Reprinted with permission from Ref. [27]. Copyright 2021 Elsevier. (**h**) Intelligent self-protected waterborne lithium-ion batteries. Reprinted with permission from Ref. [45]. Copyright 2022 John Wiley and Sons. (**i**) Temperature sensors based on seaweed sodium alginate (SA), acrylamide (AM), and iron chloride as raw materials. Reprinted with permission from Ref. [46]. Copyright 2023 Elsevier. (**j**) A flame-retardant encapsulated LED array with patterned circuits was lit at 50% strain. Reprinted with permission from Ref. [47]. Copyright 2024 John Wiley and Sons.

In recent years, hydrogel used in firefighting has attracted increasing interest due to its excellent performance, including high water contention [36], ecofriendly nature, and the availability of materials. However, relative research concerning the application of hydrogel in the firefighting field is still relatively few, and a significant research gap still exists in the firefighting field [37]. This paper seeks to provide a comprehensive review of the development of hydrogel for fire extinguishing and prevention in the last ten years. The utilization of hydrogel in the realm of fire prevention and suppression primarily encompasses three pivotal areas: hydrogel-based extinguishing agents, fire-resistant fibers, and the integration of cutting-edge fire detection systems (Figure 7). Initially, hydrogel extinguishants were developed to test their effect on solid fires, which exhibited suitable applicability in suppressing solid fires.

From as early as 2014, Yang et al. prepared the P(NIPA-co-SA) temperature-sensitive hydrogel, which can effectively extinguish a solid fire. Furthermore, the mechanism of thermosensitive hydrogel was proposed [38]. Hydrophilic groups in the molecular chain form a hydrate structure with water and are soluble in water under low temperatures. Hydrophobic groups play a leading role when temperature-sensitive hydrogel touches high-temperature objects. Subsequently, a series of porous hydrogels have been developed due to their excellent performance, including flame retardancy, flexibility, low density, compressibility, and other features, enabling their application in various fields. In 2015, Hu et al. synthesized authigenic gas foaming hydrogel, free-radical polymerization, and chemical foaming methods that were used to synthesize microporous hydrogel for controlling spontaneous combustion of coal, offering a novel methodology for addressing the issue of coal spontaneous combustion [39]. Porous structure aerogels were also proposed in 2016. Liu et al. developed highly compressible three-dimensional graphene aerogels with anisotropic porous structure, which are fabricated by directional freezing of graphene hydrogel using anisotropically grown ice crystals as templates followed by freeze-drying [40]. Similarly, He et al. prepared a multifunctional polyimide (PI) aerogels absorbent on the base of poly (amic acid) ammonium salt (PAS) hydrogels via a facile and environment-friendly method in 2017. These aerogels show excellent mechanical flexibility, fire resistance, and high thermostability, which enable them to be a highly efficient and recyclable sorbent material usable at extremely high or low temperatures [32]. Afterward, the ongoing exploration and optimization of hydrogel preparation methods and their resulting properties remain at the forefront of research. Advancements such as further improvements in fire extinguishing and flame-retardant capabilities can be achieved by altering the chemical composition, pore size, and distribution of the hydrogel. Meanwhile, by integrating hydrogel with other fire protection technologies, such as connecting them with fire warning systems, earlier and more accurate fire prevention and control can be realized. The resulting hydrogel, with excellent cooling effects, greatly reduces the time and amount of water used for fire extinguishment. Similarly, Yang et al. achieved the large-scale production of graphene aerogels through a simple physical foaming method in 2018 [41]. With microbubbles as templates, the surfactant foaming sol–gel method was used to effectively destroy and reconstruct inherent graphene oxide liquid crystals. The resultant graphene aerogels achieved a compression strain elasticity of 99% and an ultralow density of 2.8 mg/cm^3^ while also possessing fast solar thermal energy conversion and efficient fire-resistant insulation performance. Similarly, Yang et al. used a simple wet pressure assembly strategy to obtain an infinite macroscopic scale super-strong and super-elastic graphene aerogel achieved a compressive strength of 47 MPa in 2019, an ultrahigh elasticity of 97%, and a conductivity of 378 S∙m^−1^. This aerogel excels as a high-performance, fire-resistant insulation material, finding diverse applications in fire-resistant fabrics, flame-resistant protective coatings, thermally responsive membranes, and sensors, among others [42]. Similar to graphene aerogels, silica aerogels also possess excellent flame retardancy and fire resistance properties. Yu et al. reported an aerogel produced by the copolymerization of phenol-formaldehyde resin (PFR) and SiO_2_, which exhibits excellent fire resistance and can withstand high-temperature flames without disintegration [43]. A great deal of studies on hydrogel use as fire-resistant materials have been reported since 2020 due to the inherent excellent flame-retardant performance of hydrogel. Hsiao et al. integrated multi-walled carbon nanotubes (MWNTs) into a gelatin solution by using glutaraldehyde as a crosslinker [44]. With the addition of glutaraldehyde, the viscosity of the MWNT–gelatin dispersion increased with time and achieved a viscous precursor paste for conductive hydrogel using various scalable coating techniques, including blade coating. After large-area printing, the MWNT–gelatin paste continued to crosslink and obtain MWNT-integrated gelatin hydrogel (MW–hydrogel). The MW–hydrogel was highly deformable, and the resistance of conductive MW–hydrogel responded to various mechanical deformations, enabling their use in electronic robot skin for real-time monitoring of soft robot actuation. In addition, the MW–hydrogel was further used as flame-resistant skin for soft robot grippers that can handle and rescue irregular objects from fire scenes. In 2021, Yu et al. prepared a new type of three-dimensional network hydrogel that can effectively achieve flame retardancy when layered on fabric [27]. The fabric remained intact when exposed to flames for 12 s. Moreover, it exhibited excellent antibacterial and swelling properties. Yang et al. prepared a thermally responsive membrane by in situ polymerization on a hydrophilic film in 2022 [45]. Intelligent self-protected waterborne lithium-ion batteries were developed successfully. This should be attributed to the thermally responsive membrane blocking the lithium-ion transmission channels at high temperatures and reopening them when cooled, and this control is reversible. In 2023, Zhang et al. prepared temperature sensors based on seaweed sodium alginate (SA), acrylamide (AM), and iron chloride as raw materials with temperature sensitivity from 25 to 90 °C with a resolution of 1.28%/°C. The resulting sensors can be used for high-resolution detection of temperature changes with excellent flame retardancy and flexibility. Moreover, the introduction of water-soluble glycerol enhanced its water retention performance [46]. A hydrogel encapsulation strategy was proposed to enhance the flame retardancy and thermal stability of stretchable electronics in 2024 [47]. Hydrogel-based encapsulation provides thermal protection against flames for more than 10 s through the evaporation of water. The incorporation of hydrogel encapsulation enables stretchable electronics to maintain their functions and perform complex tasks, such as fire saving in soft robotics and integrated electronics sensing.

Overall, the application of hydrogel in the field of firefighting holds vast prospects and potential. In the future, with deeper research into the fire extinguishing mechanisms and properties of hydrogel, as well as the continuous optimization of preparation techniques, it is believed that hydrogel will play a greater role in the firefighting field, making significant contributions to safeguarding people’s lives and property.

## 3. Types of Hydrogel Extinguishants

### 3.1. The Characterizes of Different Hydrogel Extinguishants

Hydrogel extinguishants can be categorized into several types according to recent studies: thermosensitive hydrogel extinguishants, hydrogel foam extinguishants, hydrogel dry water extinguishants, and common hydrogel extinguishants. A summary and comparison of the different hydrogel extinguishants are presented in Table 1. The most prominent feature of thermosensitive hydrogel is their ability to undergo a reversible sol–gel phase transition at a certain temperature, which is known as the lower critical solution temperature (LCST) [39,48]. The thermosensitive hydrogel extinguishants exhibit low viscosity and high fluidity at room temperature, making them highly transportable. However, hydrophobic association occurs at high temperatures (such as 80 °C) due to the weakening of hydrogen bonding, leading to physical crosslinking and the formation of a gel. This hydrogel possesses strong adhesiveness, enabling it to adhere to the surface of combustible materials for extended periods, effectively blocking oxygen and maintaining a reduced temperature. Interestingly, it returns to its original sol state once the fire is extinguished and the temperature drops. The reversibility of this sol–gel transition minimizes the environmental impact of this fire extinguishing material. Consequently, the thermosensitive hydrogel extinguishants serve as an intelligent, stimulus-responsive material, possessing exceptional benefits in the realm of fire suppression. Hydrogel foam extinguishants refer to a class of fire-suppression agents formulated with gel foam. In comparison to other hydrogel extinguishants, hydrogel foam extinguishants stand out for oil fires as they swiftly create a dense water film on the liquid’s surface, effectively achieving rapid cooling and oxygen isolation, thus offering distinct advantages. Compared with traditional foam extinguishants, the formation mechanisms of foam are different, even if both belong to water-based extinguishants. Foam generation of traditional foam extinguishants is mainly attributed to chemical reactions, while hydrogel extinguishants rely on surfactants through physical methods. In addition, the gel foam of hydrogel foam extinguishants has a more stable structure, significantly improving its fire resistance. Hydrogel dry water extinguishants refer to extinguishants that combine the advantages of dry powder and water mist with a water content of over 90%. The introduction of hydrogel significantly enhances the core–shell structure of dry water, enabling it to sustain a robust and stable core–shell configuration [49,50,51] during the fire extinguishing process. The large specific surface area and high water content of hydrogel dry water extinguishants endow it with high-efficiency fire extinguishing performance for Class A fires. The large specific surface area allows for a greater contact area with the fire source, enhancing the cooling effect and fire-suppression capabilities. Meanwhile, the high water content ensures a sufficient supply of water to effectively extinguish the fire. Together, these properties make hydrogel dry water extinguishing agents an effective choice for tackling Class A fires. Common hydrogel extinguishants refer to a type of extinguishing agent characterized by high water content, adhesion, non-foaming properties, temperature insensitivity, and a lack of a core–shell structure. This type of hydrogel extinguishants is widely studied in the field of suppressing spontaneous combustion in coal mines [52,53,54,55,56,57,58,59,60,61,62].

### 3.2. Critical Raw Materials

Radical copolymerization and ionic crosslinking stand as the two primary crosslinking modalities. The realm of gel materials encompasses both natural polymeric materials, such as sodium alginate (SA) and cellulose derivatives (MC and CMC), as well as synthetic polymeric materials, exemplified by polyethylene glycol (PEG) and N-isopropylacrylamide (NIPAM). It is also reported in the relevant literature that other gelling agents such as modified polyethoxated silicone (MPS), 2-Acrylamide-2 methyl propane sulfonic acid (AMPS), sodium polyacrylate (PAAS), chitosan (CS), Xanthan gum (XG), etc., are used in hydrogel extinguishants. Among these gelling agents, NIPAM is particularly notable. Its polymer, poly(N-isopropylacrylamide) (PNIPAM), exhibits excellent thermosensitive properties and film-forming ability, which is crucial for suppressing fire. Alginate sodium, as a natural polymer material, not only possesses excellent biodegradability, demonstrating a high degree of environmental friendliness, but also maintains suitable thermal stability under high-temperature conditions. Cellulose, on the other hand, occupies a unique position in fire extinguishing agents due to its strong adhesive properties and favorable cost-effectiveness. Polyethylene glycol (PEG), with its suitable compatibility and moisturizing properties, can seamlessly integrate with various components to enhance the effectiveness of fire extinguishing. All these unique properties are significant factors for the application of alginate sodium, cellulose, and polyethylene glycol in the field of fire extinguishing agents. Organic crosslinking agents are primarily N, N’-Methylenebisacrylamide (MBA), while inorganic crosslinking agents are mostly magnesium or calcium salts, such as magnesium or chloride calcium chloride. The initiator is ammonium persulfate (APS) or potassium persulfate (KPS). A summary and comparison of the critical raw materials of thermosensitive hydrogel extinguishants are presented in Table 2.

In contrast to thermosensitive hydrogel extinguishants, hydrogel foam extinguishants incorporate additional foaming agents into their raw materials, giving the extinguishants the characteristics of low density and strong wettability. The foaming agents are primarily one or more surfactants, commonly used ones being sodium laureth sulfate (AES), alkyl polyglucoside (APG), sodium dodecyl sulfate (SDS), sodium dodecyl benzene sulfonate (SDBS), and sodium alpha-olefin sulfonate (AOS). Alkyl polyglycosides are widely used in water-based fire extinguishing agents due to their low surface tension, resistance to strong acids and bases, low toxicity, low irritancy, and complete biocompatibility. More importantly, they can produce fine and abundant foam, achieving significant synergistic effects and compatibility with other surfactants. There are also relevant literature reports on other surfactants, such as hydrolyzed protein (HP), hexadecyl trimethyl ammonium bromide (CTAB), sodium dodecyl sulfonate (SLS), etc., being used as foaming agents for hydrogel fire extinguishing agents. The main crosslinking method is ionic crosslinking, with calcium chloride and organic aluminum salts (PAC) as the primary crosslinking agents. The critical raw materials of hydrogel foam extinguishants are summarized in Table 3. The main components of hydrogel dry water extinguishants are water and silica. Relevant studies have shown that adding gel can enhance the core–shell structure of dry water, achieving better fire extinguishing effects. Dry water typically contains over 90% water content, and its water content can be adjusted to meet fire extinguishing needs by varying the solid-to-liquid ratio. The critical raw materials of hydrogel dry water extinguishants are summarized in Table 4.

### 3.3. Preparation Strategies

**Figure 8:**
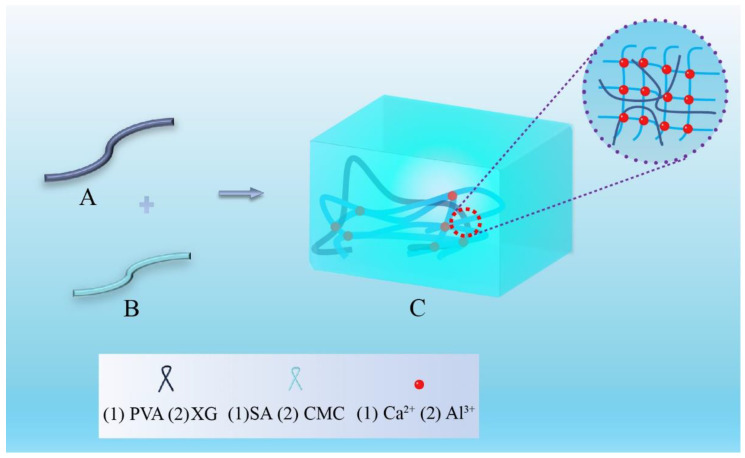
The preparation of hydrogel extinguishants by ionic crosslinking.

**Figure 9:**
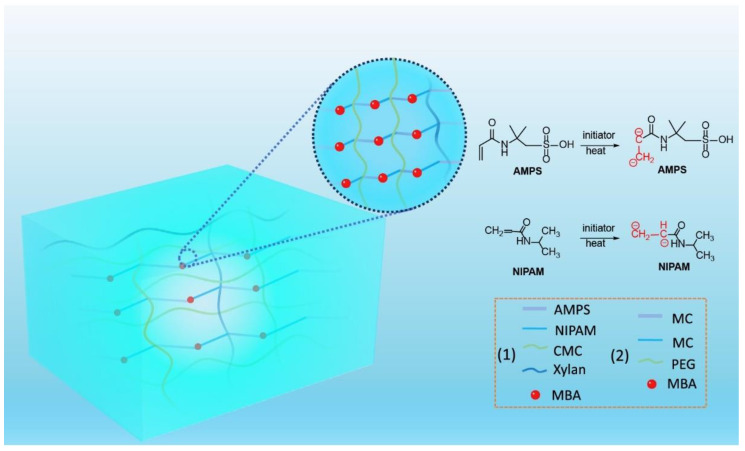
The preparation of hydrogel extinguishants by free-radical copolymerization.

**Figure 10:**
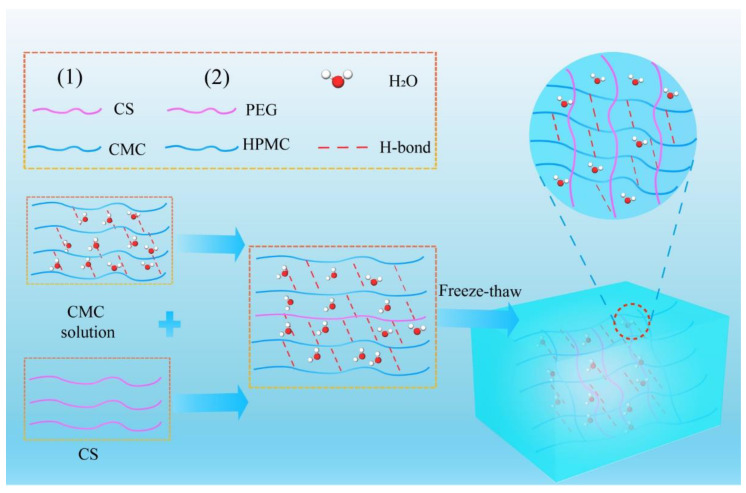
The preparation of hydrogel extinguishants by hydrogen bond crosslinking.

In the preparation strategies of hydrogel extinguishants, the gelation mechanisms of hydrogel involve three common crosslinking methods: ionic crosslinking (Figure 8), radical copolymerization (Figure 9), and hydrogen bond crosslinking (Figure 10). Ionic crosslinking involves introducing ionic crosslinking agents into the material, allowing them to interact with ions in the material to form strong chemical bonds, thereby improving the physical and chemical properties of the material. In hydrogels, ionic crosslinking agents are typically charged ions such as calcium, iron, and aluminum. These ions interact with ionic groups (such as carboxyl and sulfonic acid groups) on the polymer chains of the hydrogel, forming an ionic bond crosslinking network [77]. Ionic crosslinked hydrogels exhibit high stability and mechanical strength [78], and their properties can be tailored by adjusting the type and concentration of the ionic crosslinking agent. Additionally, ionic crosslinked hydrogels possess suitable biocompatibility and biodegradability.

Free-radical copolymerization is a chemical crosslinking method that utilizes light, heat, radiation energy, or an initiator to activate several monomer molecules into free radicals and induce copolymerization. During free-radical copolymerization, the monomer molecules connect with each other through covalent bonds, forming polymer chains, which in turn construct the three-dimensional network structure of the hydrogel. Free-radical copolymerization reactions possess high controllability and flexibility, allowing for the customization of hydrogel properties by selecting different monomers and initiators according to specific needs. Hydrogen bond crosslinking is a physical crosslinking method that primarily relies on the hydrogen bond interactions between polar groups on the polymer chains of the hydrogel [79,80,81,82,83]. In hydrogel, polar groups (such as hydroxyl and amino groups) on the polymer chains can form hydrogen bonds, constructing the three-dimensional network structure of the hydrogel. Hydrogen bond crosslinked hydrogels possess reversibility and self-healing properties [84]. Processing methods include ultrasonic treatment, solvent substitution, microwave foaming, acid–heat treatment, freeze–thaw cycling, grinding, vacuum-drying, and others.

**Figure 11:**
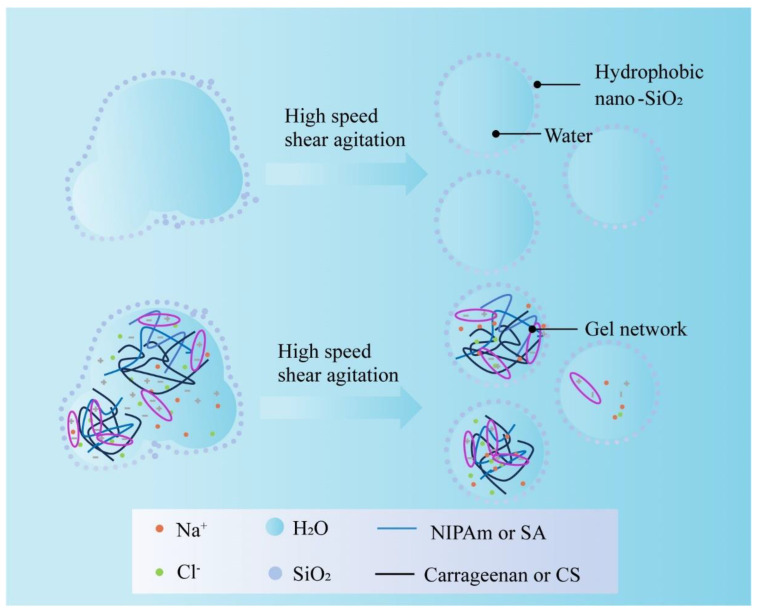
The preparation of hydrogel extinguishants with dry water core–shell structure.

However, the processing methods for hydrogel dry water extinguishants are rather unique (Figure 11). This involves high-speed shear stirring, where the energy generated changes the surface of hydrophobic silica and the solution, allowing the hydrophobic silica to adhere to the surface of the droplets, improving their wetting properties. Part of the silica is wetted to form a core–shell structure [76]. Additionally, the introduction of gel enhances the core–shell structure of dry water, improving its fire extinguishing performance.

Overall, the preparation of hydrogel extinguishants can be divided into two strategies. The first method involves vacuum-drying the prepared components and then grinding them into a powdered substance. When needed, the powdered substance is soaked in an aqueous solution to allow it to fully swell, and it can then be used as a hydrogel extinguishant. This method of preparing hydrogel extinguishants is more convenient for storage, but the steps are slightly cumbersome. This solid powder hydrogel fire extinguishant necessitates rigorous regulation of its particle size, coupled with exceptional rapidity in swelling and formidable water absorption capabilities, thereby facilitating swift water absorption and expansion for fire suppression in times of need. Presently, certain commercially available hydrogel fire extinguishants, when mixed with water in precise proportions, can promptly generate a gel in less than 3 min, attaining a remarkable water absorption ratio ranging from 400 to 750 times. After spraying, the gel coating can isolate air, suppress the diffusion of toxic and harmful gases, lower the temperature of the burning surface, and effectively prevent the spread of fire. The second way directly involves dissolving a certain number of raw materials, including crosslinking agents and gelling agents, in specific proportions under suitable conditions. Finally, the components are mixed, and a gelation reaction occurs at a specific temperature. Depending on the actual needs, catalysts, foaming agents, flame retardants, etc., can be added after mixing. Commonly used catalysts are N, N, N’, N’-Tetramethylethylenediamine (TEMED), and flame retardants include inorganic flame retardants and organic flame retardants. Common inorganic flame retardants refer to hydroxides such as aluminum hydroxide and magnesium hydroxide; inorganic phosphorus-based flame retardants, such as ammonium phosphate and ammonium polyphosphate; and nitrogen-based flame retardants, such as urea. Organic flame retardants include phosphate esters, melamine, and some halogenated flame retardants. It is noticed that both their effectiveness and environmental considerations should be taken into account when using these flame retardants. Here are some specific cases for reference. Studies have shown that introducing macroporous organic lignin can improve the thermal stability of hydrogel extinguishants. Firstly, NIPAM, acrylic acid (AA), lignin, and MBA are dissolved in deionized water separately. After complete dissolution, the components are mixed and continuously stirred. Then, an initiator APS and a catalyst TEMED are added, along with an appropriate amount of deionized water. Finally, the polymerization reaction is allowed to proceed for 20 min at room temperature. The successfully prepared hydrogel extinguishants can effectively control the spontaneous combustion of coal [60]. An additional scenario involves utilizing gelling agents such as sodium acrylate and methylcellulose, in conjunction with magnesium chloride as a crosslinking agent, to create thermosensitive hydrogel extinguishants through a straightforward mixing process specifically designed for application in Class A fires.

### 3.4. Evaluation System of Hydrogel Extinguishants

#### 3.4.1. Water-Based Extinguishants

Physical and chemical properties and fire extinguishing performance are two parts of the evaluation system for water-based fire extinguishants according to GB 17835-2008 “Water-based Fire extinguishing Agents”. Physical and chemical properties refer to freezing point, freeze–thaw resistance, pH, surface tension, corrosion rate, and toxicity. Fire extinguishing performance includes the ability to extinguish Class A and Class B fires. According to relevant requirements, the freezing point of water-based fire extinguishants should be controlled within the range of −4~0 °C and the pH value within 6.0~9.5. The corrosion rate on steel or aluminum sheets should be less than 15.0 mg/(d·dm^3^), and the mortality rate of fish should not exceed 50%. Regarding fire extinguishing performance for Class B fires, the fire extinguishing rating must be at least 55 B when rubber industry solvent oil is involved, and for 99% acetone, the rating should meet or exceed 34 B. For Class A fires, the fire extinguishing rating requirement is greater than or equal to 1 A. Surprisingly, despite our knowledge, no researcher has yet conducted comprehensive evaluation tests on hydrogel extinguishants in strict adherence to national standards. Even more concerning, corrosion rate and toxicity tests remain unperformed, posing a significant obstacle to the commercialization of these agents. Most studies have only focused on fire extinguishing performance tests while rarely involving physical and chemical properties such as freezing point, resistance to freeze–thaw cycling, toxicity, and corrosion rate. Overall, in the current research on hydrogel extinguishants, the evaluation system mainly includes microscopic characterization and fire extinguishing performance testing, which neglects the national requirements for ecological environmental protection and sustainable development compared to the national standards for water-based fire extinguishants. The low toxicity requirement in the national standards for water-based fire extinguishants is straightforward to fulfill, as most natural high-molecular materials used in the preparation of hydrogel extinguishants are low-toxic or non-toxic. However, ensuring a low corrosion rate while maintaining satisfactory fire extinguishing performance poses a greater challenge. This necessitates the incorporation of corrosion inhibitors during the manufacturing process. Commonly used corrosion inhibitors include triethanolamine, hexadecyl amine, sodium gluconate, etc.

#### 3.4.2. Hydrogel Extinguishants

**Figure 12:**
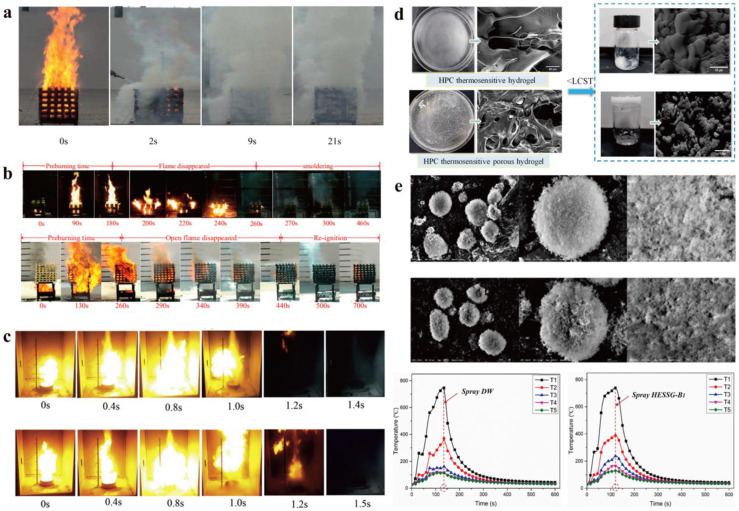
The evaluation system of hydrogel extinguishants: (**a**) Aerogel fire extinguishing agent used to carry out fire extinguishing experiments of Class A standard fire. Reprinted with permission from Ref. [85]. Copyright 2023 Elsevier. (**b**) Hydrogel extinguishants for extinguishing fire compared with foam extinguishants. Reprinted with permission from Ref. [66]. Copyright 2014 MDPI. (**c**) Hydrogel extinguishants for Class B fires. Reprinted with permission from Ref. [74]. Copyright 2019 Elsevier. (**d**) Microporous structure of thermosensitive porous hydrogels. Reprinted with permission from Ref. [86]. Copyright 2023 MDPI. (**e**) SEM images of HSESG before and after fire suppression. Reprinted with permission from Ref. [87]. Copyright 2021 Elsevier.

Among various hydrogel extinguishants, the common evaluation methods primarily include the testing of fire-suppression performance for Class A and Class B fires, as well as microscopic characterization before and after extinguishing. To demonstrate that hydrogel extinguishants exhibit better extinguishing efficiency for Class A fires compared to water, some researchers tested the extinguishing effect of water and hydrogel extinguishants on a 1 m^3^ wood crib fire under identical conditions. According to national standards, a 1 m^3^ wood crib refers to specially treated wooden logs with a diameter of 40 ± 1 mm and a length of 500 ± 10 mm, stacked in layers with equal spacing, with 7 logs per layer and 12 layers in total. In the process of fire suppression, a large amount of water fails to truly function effectively due to its strong fluidity [38]. In contrast, the adhesive properties of hydrogel extinguishants enable them to form a dense film on the surface of combustible materials, ensuring the isolation of oxygen while also providing a continuous cooling effect. Therefore, hydrogel extinguishants can achieve better fire-suppression results. Liu et al. [85] developed an aerogel fire extinguishing agent, which was used to carry out fire extinguishing experiments of Class A standard fire. When the fire extinguishing reached about 9 s, the open fire of the wooden stack basically disappeared, and the fire extinguishing experiment lasted until 21 s (Figure 12a). Additionally, relevant studies have also shown that hydrogel extinguishants are faster and more efficient in extinguishing fire compared with foam extinguishants (Figure 12b) [66]. Here, a comparison was made between the fire-suppression effects of thermosensitive hydrogel extinguishants, foam extinguishants, and ordinary hydrogel extinguishants. Hydrogel extinguishants can better prevent reignition compared to foam extinguishants. Experimental results indicate that the extinguishing time for ordinary hydrogel extinguishants and foam extinguishants is 340 s and 240 s, respectively, while the thermosensitive hydrogel fire extinguishing agent requires only 120 s. Hydrogel extinguishants also exhibit suitable extinguishing effects for Class B fires (Figure 12c) [74]. Before performing extinguishing, the gasoline in the oil pan needs to be ignited for 60 s to allow it to burn fully, and 99% acetone fire needs to pre-burn for 120 s. Then, thermosensitive hydrogel extinguishants and hydrogel extinguishants are used for extinguishing. According to national standards, a certain amount of fuel, including 2/3 gasoline and 1/3 water, is added to the oil pan. Three parallel fire extinguishing tests are conducted, and if two of them are successful, the requirements are met. It is worth noting that the fire-suppression performance of hydrogel extinguishants is closely related to their microscopic structure. Ma et al. [86] prepared thermosensitive porous hydrogels (Figure 12d) and characterized their microporous structure at the microscopic level, which endows the hydrogel with excellent water absorption and retention properties. Similarly, some studies have also demonstrated that a stable hydrogel structure is one of the factors to consider for fire-suppression effectiveness. By comparing the microstructures of hydrogel dry water extinguishants before and after fire suppression, it can be found that although the hydrogel enhances the dry water structure, high-temperature conditions still lead to the rupture and shrinkage of the hydrogel extinguishants, causing the leakage of core materials (Figure 12e) [87]. Additionally, the temperature change during the fire-suppression process was monitored using thermocouples, and the results showed that compared to ordinary dry water, the use of hydrogel dry water extinguishants enables faster temperature reduction.

**Figure 13:**
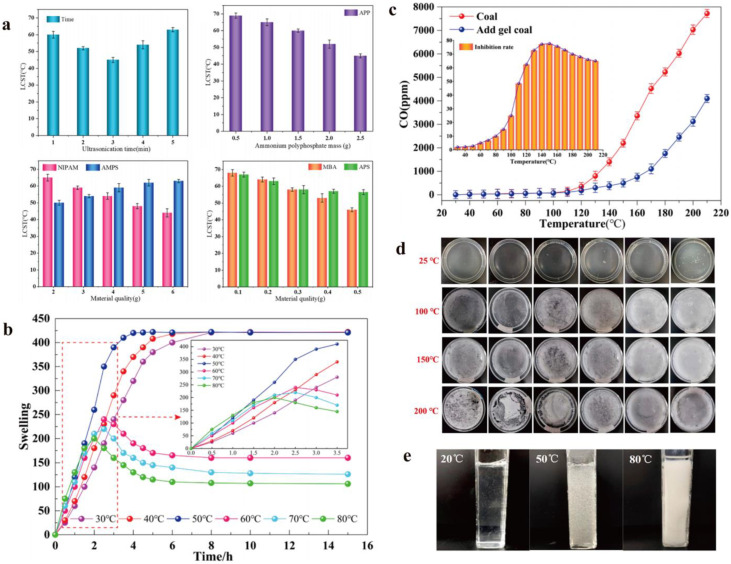
The evaluation system of thermosensitive hydrogel extinguishants: (**a**) The LCST of thermosensitive hydrogel extinguishants in different situations. (**b**) Expansion rate of the hydrogel extinguishants varies at different temperatures. (**c**) The suppression rate of carbon monoxide. Reprinted with permission from Ref. [64]. Copyright 2022 Elsevier. (**d**) The thermal stability of hydrogel fire extinguishants with different concentrations. Reprinted with permission from Ref. [86]. Copyright 2023 MDPI. (**e**) Hydrogel fire extinguishants undergo a sol–gel phase transition at 50–80 °C. Reprinted with permission from Ref. [66]. Copyright 2021 MDPI.

The most typical evaluation of thermosensitive hydrogel extinguishants is the phase transition temperature, also known as the lower critical solution temperature (LCST). The LCST value is related to the raw materials used in preparing the hydrogel extinguishants, such as the type and concentration of the polymer [64], and the preparation conditions, including temperature, pressure, and pH, can also have an impact on it (Figure 13a). However, the fundamental reason ultimately lies in the proportion of hydrophilic and hydrophobic groups in the polymer material. In addition, the expansion rate of the hydrogel extinguishants and the suppression rate of carbon monoxide also have significant effects on the firefighting performance (Figure 13b,c). As shown in the figure, the expansion rate of the hydrogel extinguishants varies at different temperatures, with a maximum expansion rate of up to 450 times [65]. At temperatures ranging from 30 to 50 °C, the solution is in a sol state, exhibiting a faster increase in water absorption rate and a longer swelling equilibrium time. At 60~80 °C, the water absorption rate increases, and the time to reach swelling equilibrium shortens. This is because as the temperature rises, hydrophilic groups such as hydroxyl and carboxyl groups become more active, and the wrinkled state resulting from shrinkage also helps it absorb a large amount of water. Furthermore, compared to no hydrogel extinguishants, the production of carbon monoxide from coal combustion is significantly reduced, indicating that hydrogel extinguishants can effectively inhibit the generation of carbon monoxide. Ma et al. explored the thermal stability of hydrogel fire extinguishants with different concentrations (Figure 13d). The results showed that when the concentration is below 15%, due to the evaporation of water, the entire system quickly becomes a dry gel film with an uneven morphology at 200 °C [86]. However, when the concentration is greater than 15%, the system remains in a relatively stable state, maintaining a wet gel film even after 600 s at 200 °C. This thermal stability is crucial for the firefighting performance of the hydrogel extinguishants. It is worth noting that no studies have proven the optimal phase transition temperature for thermosensitive hydrogel fire extinguishants. Considering various factors, we believe that 80 °C is a suitable choice. As shown in Figure 13e, the hydrogel extinguishant undergoes a sol–gel phase transition at 50–80 °C, exhibiting higher viscosity and lower surface tension [66]. This helps it adhere to the surface of combustibles, achieving the effect of isolating oxygen and continuously reducing temperature.

**Figure 14:**
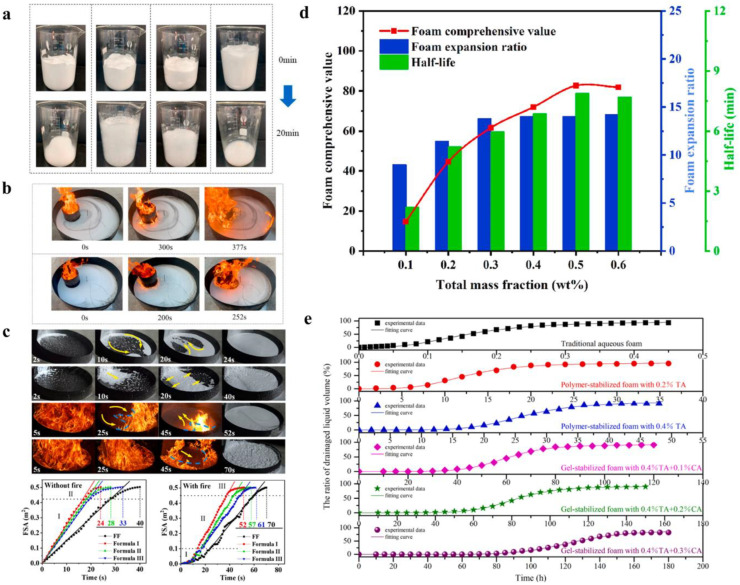
The evaluation system of hydrogel foam extinguishants: (**a**) Stability of gel protein foam extinguishants. (**b**) The burn resistance of hydrogel foam extinguishants. Reprinted with permission from Ref. [70]. Copyright 2023 Elsevier. (**c**) Spreading ability on fuel surfaces of hydrogel foam extinguishants. Reprinted with permission from Ref. [68]. Copyright 2024 Elsevier. (**d**) The overall performance of the foam. Reprinted with permission from Ref. [70]. Copyright 2023 Elsevier. (**e**) The liquid drainage time of hydrogel foam fire extinguishants. Reprinted with permission from Ref. [73]. Copyright 2022 Elsevier.

In the evaluation system of hydrogel foam extinguishants, the stability of the foam is particularly crucial [70]. Related studies have shown that compared to aqueous film-forming foam (AFFF) extinguishants, gel protein foam extinguishants possess better foam stability due to the hydrogel’s enhancement of the foam’s structural stability (Figure 14a).

This is groundbreaking for the research on hydrogel foam fire extinguishants. In addition, the burn resistance (Figure 14b) [70] and spreading ability on fuel surfaces (Figure 14c) [68] of hydrogel foam extinguishants are also worthy of attention, as these properties directly impact their firefighting performance. The burn resistance of hydrogel foam fire extinguishants is of significant value in extinguishing liquid fires, as a foam with suitable burn resistance can effectively extinguish the fire while preventing reignition. The burn resistance test mainly includes three typical stages: stable coverage, partial collapse, and diffusive collapse. In the test of foam spreading performance, the foam spread area graph (FSA) indicates that the spreading process can be divided into two stages: a stable growth phase and a slow growth phase. Compared to non-burning tests, the foam spreading speed increases faster in the first stage of the burning test. The ability of the foam to quickly spread on the fuel surface and maintain stable burn resistance are two crucial factors for hydrogel foam fire extinguishants to extinguish liquid fires. To screen for the optimal mass fraction of the foaming agent, the foam comprehensive index (FCI) is often used to evaluate the overall performance of the foam (Figure 14d). This value is calculated based on the foam’s half-life and expansion ratio, and research shows that a 6% foaming agent concentration yields suitable overall foam performance [70]. Furthermore, the liquid drainage time [73] is also a key parameter in evaluating the performance of hydrogel foam fire extinguishants (Figure 14e).

**Figure 15:**
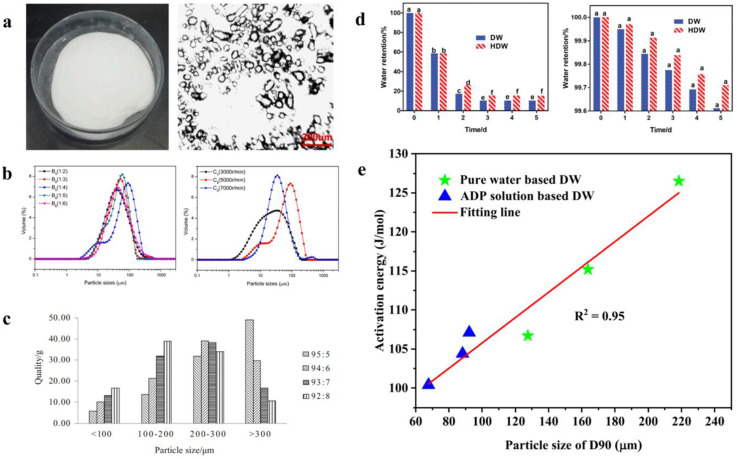
The evaluation system of hydrogel dry water extinguishants: (**a**) The morphology of dry water. (**b**) The particle size distribution of dry water. Reprinted with permission from Ref. [87]. Copyright 2021 Elsevier. (**c**) Effect of solid–liquid ratio on particle size of dry water. Reprinted with permission from Ref. [49]. Copyright 2020 Springer Nature. (**d**) The water retention performance of dry water extinguishants. Reprinted with permission from Ref. [75]. Copyright 2023 Taylor and Francis. (**e**) Effect of particle size of dry water on activation energy. Reprinted with permission from Ref. [88]. Copyright 2024 Elsevier.

The evaluation system of hydrogel dry water fire extinguishants mainly encompasses micro-morphology, particle size distribution, water content, activation energy, and other aspects. Dry water is a powdery substance (Figure 15a), and under an optical microscope, one can clearly observe small water droplets encapsulated by silica, resembling tiny bubbles [87]. The particle size of dry water is mainly concentrated between 10 and 300 μm. As depicted in Figure 15b, the stirring speed has a significant impact on its particle size distribution, while the solid–liquid ratio has a relatively smaller effect. Related studies have also shown that the stirring time is also a factor affecting its particle size distribution. Han et al. studied the particle size distribution of hydrogel dry water fire extinguishants under different solid–liquid ratios and concluded that as the solid–liquid ratio increases [49], the particle size decreases (Figure 15c). It is proved that when the particle size is between 10 and 300 μm, the fire extinguishing effect is optimal [73,87]. The water content and water retention properties of hydrogel dry water fire extinguishants have a direct relationship with their firefighting performance. Dou et al. studied the water retention properties of ordinary dry water and hydrogel dry water. The results showed that hydrogel dry water exhibits better water retention properties than ordinary dry water [75], both in open and enclosed environments (Figure 15d). Additionally, the particle size also directly affects its activation energy [88], with an increase in particle size leading to a decrease in activation energy (Figure 15e).

## 4. Commercial Aspects of Hydrogel Extinguishants

### 4.1. Commercialization Situation of Hydrogel Extinguishants

In recent years, the commercialization of hydrogel fire extinguishants has also made a series of major breakthroughs. A fire protection company in Sichuan has developed a polymer gel fire extinguishant that can effectively extinguish Class A fires. The extinguishant is a powdered substance that can be mixed with water at a ratio of 1:300 or 1:500 to form an environmentally friendly and highly effective extinguishant. At high temperatures, it forms a stable coating that effectively blocks heat conduction, instantly extinguishes fires, and has flame retardance, cooling, and efficacy that are 8 to 13 times that of traditional products. In the same way, the company has also developed a gel protective device that can withstand a high temperature of 1200 °C for 120 s, which can be simply sprayed on people. The resin gel additive produced by another fire protection company can be mixed with water to form a hydrogel fire extinguishing liquid within 3 min at the earliest. The fire extinguishing liquid has no pollution to soil and suitable environmental protection performance. The research and development of foreign hydrogel materials and products are concentrated in the fields of aerospace, naval ships, and other military fire protection, with high prices and relatively limited commercialization. In response to the risk of steam pipe rupture and sudden temperature rise in nuclear submarines under deep-diving conditions, the U.S. Navy’s Naval Sea Systems Command (NAVSEA) and clothing and textile research institutions have developed a new protective suit. In the international market, the hydrogel fire blanket developed by First Aid Only of the United States can help extinguish the flame and cool the burn, help stabilize the emotions of the burn patients, and assist in the treatment of emergency. Compared with other types of fire extinguishants, hydrogel fire extinguishants have high-cost performance and suitable fire extinguishing performance, which can effectively improve the actual fire extinguishing efficiency of first-line fire rescue personnel and can minimize the damage to the ecological environment around the building on fire. Therefore, hydrogel fire extinguishants have greater development advantages in the fire extinguishant market. In the long-term research process, the application performance of this type of fire extinguishants has been continuously optimized, and it still has suitable fire extinguishing effects in practical fire extinguishing tests under various types of fires and ultralow temperatures. It can fully become an effective tactical measure for firefighting personnel at all levels in China to handle fires. The commercial applications of hydrogel extinguishants in various fields have tremendous potential. In recent years, research on the use of hydrogel extinguishants in fighting forest, grassland, building, liquid pool, coal mine, and lithium-ion battery fires has garnered significant attention [28,89,90]. However, in practical application, the use of hydrogel fire extinguishants is restricted by factors such as production process, formula cost, storage conditions, and spraying equipment, which also leads to problems such as more types of hydrogel fire extinguishants research and development and a limited number of widely used hydrogel fire extinguishants. There is still great room for development in the future.

### 4.2. The Future Development Trend of Commercial Hydrogel Extinguishants

(1) Expanding the use of hydrogel extinguishants. Hydrogel extinguishants have the advantages of wide sources of materials and low cost due to the water being the main component of hydrogel, which provides natural, convenient conditions for production. The scope of its use can be expanded by improving the formula and process to carve out a larger market.
(2) Promoting advanced auxiliary equipment. To better apply hydrogel extinguishants, equipment technology needs to be improved to maximize its efficiency and reduce limitations in terms of storage, transportation, and equipment functionality. For example, an automatic premix device for extinguishants before spraying can be designed to improve the extinguishing performance of water in water supply networks or fixed tanks, thereby expanding the synthesis and application of hydrogel extinguishants. In addition, the research on the atomization mechanism can be further deepened, and new atomization nozzles can be innovatively developed to improve the spraying effect.
(3) Promoting multidisciplinary integration. To better study hydrogel extinguishants, it is necessary to promote multidisciplinary integration. Biological science can be used to find microorganisms with specific properties suitable for hydrogel extinguishants to reduce the pressure on environmental protection.
(4) Promoting the development of standardization. To lead the development direction of hydrogel extinguishants, it is necessary to establish and improve the design specifications and environmental performance evaluation system for hydrogel extinguishants in China.

## 5. Hydrogel for Fire Prevention

### 5.1. Fire Prevention Fabric

**Figure 16:**
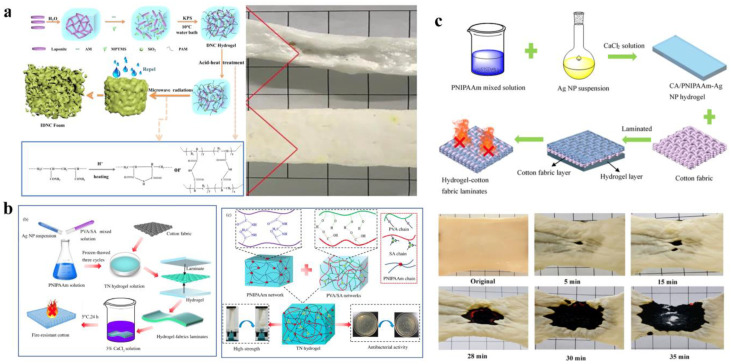
Hydrogel for fire prevention fabric: (**a**) dimensional stable hydrogel-born foam by simple acid–heat treatment and subsequent fast microwave foaming. Reprinted with permission from Ref. [92]. Copyright 2020 Elsevier. (**b**) The prepared hydrogel fireproof fabric with antibacterial properties and excellent flame retardancy by using the laminating method. Reprinted with permission from Ref. [27]. Copyright 2021 Elsevier. (**c**) Hydrogel cotton fabric with outstanding heat preservation, heat insulation, and antibacterial properties. Reprinted with permission from Ref. [35]. Copyright 2021 Springer Nature.

With its unique advantages, hydrogel has demonstrated remarkable performance in fire extinguishing. Surprisingly, it also gained widespread application in the field of fire prevention, covering diverse areas such as fireproof fabrics [91,92,93,94,95,96,97] and fire warning systems [46,98,99,100]. Currently, laminating, microwave foaming, and wet spinning are the two most used strategies in the preparation of hydrogel fireproof fabrics. For instance, Xu et al. [92] fabricated a dimensional stable hydrogel-born foam by simple acid–heat treatment and subsequent fast microwave foaming. This kind of foam avoids pore collapse in both the preparation and use process and shows excellent dimensional and thermal insulation. In another innovative study, Yu et al. [27] successfully prepared a hydrogel fireproof fabric (Figure 16b) with antibacterial properties and excellent flame retardancy using the laminating method. This fabric remained intact after being burned for up to 12 s in a combustion test. In addition, another hydrogel cotton fabric with outstanding heat preservation, heat insulation, and antibacterial properties has been developed (Figure 16c) that can effectively prevent cotton fabric from burning for 30 s at temperatures of up to 1200 °C [35]. These significant performance data fully demonstrate the outstanding performance of hydrogels in the field of fire prevention. Overall, hydrogels have made significant breakthroughs in the field of fire prevention research, demonstrating great potential and application value. Looking ahead, we have reason to believe that more intelligent and high-performance hydrogel fireproof fabrics will continue to be launched, bringing revolutionary changes to the field of fire safety.

### 5.2. Fire Warning Systems

**Figure 17:**
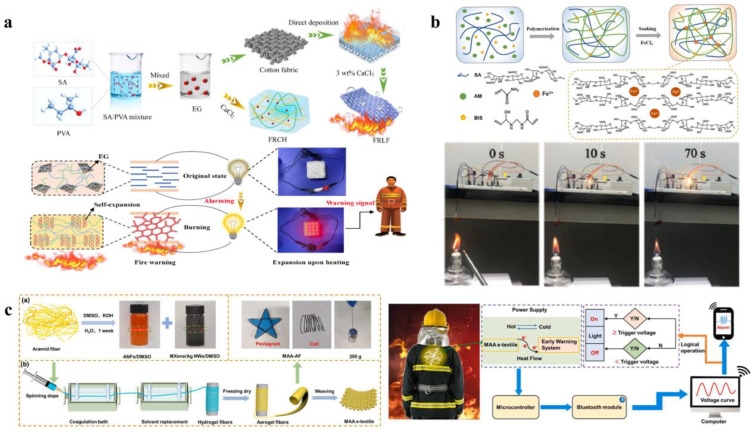
Hydrogel for fire-warning systems: (**a**) Sensors with rapid response to fires within 5 s. Reprinted with permission from Ref. [99]. Copyright 2021 Springer Nature. (**b**) Temperature-sensitive hydrogel sensor with excellent reusable performance. Reprinted with permission from Ref. [46]. Copyright 2023 Elsevier. (**c**) Wearable thermally self-powered fire alarm sensor. Reprinted with permission from Ref. [100]. Copyright 2023 Elsevier.

With the rapid development of the social economy, the application of hydrogels in fire extinguishing and prevention is gradually moving toward intelligence [35,101,102]. Currently, research on hydrogels for fire warning has garnered significant interest. For instance, Liu et al. [99] developed a novel fire-resistant composite hydrogel (FRCH) (Figure 17a) through the laminating method, which not only possesses a low thermal conductivity coefficient (0.0325 W/m∙k) but also achieves rapid response to fires within 5 s. This holds broad application prospects in fire alarm and detection under emergency fire risks. Considering both applicability and reproducibility, Zhang et al. [46] prepared a temperature-sensitive hydrogel sensor with excellent reusable performance, maintaining 90% of its sensing capability after being stored at 90 °C for 24 h (Figure 17b). Similarly, He et al. [100] carefully prepared MXene/AgNWs/ANFs (MAA) aerogel fiber-based textiles using the wet spinning strategy (Figure 17c). Based on this, they developed a wearable thermally self-powered fire alarm sensor. This sensor, when firefighters are exposed to extreme environments ranging from 100 to 400 °C, not only exhibits excellent flame retardancy (reaching 13.75 W/g) but also can promptly alert firefighters to evacuate within 1.6 s.

## 6. Conclusions: Challenges and Prospects of Hydrogel Extinguishants

Totally, remarkable research progress has been made in the development of hydrogel extinguishant materials and their application in fire protection in recent years. Some materials and products have already been used in fire suppression in forests, grasslands, buildings, mines, and other fields, demonstrating the performance advantages of hydrogel-based fire extinguishing materials in the field of fire protection. However, hydrogel fire extinguishing materials are still in their infancy, and there are many mechanisms and principles that urgently need to be explored at the fundamental level of material design. Marketed products still face challenges such as susceptibility to freezing at low temperatures, limited long-term stability, difficulty in regulating rheological properties, and finite fire extinguishing range.

To the best of our knowledge, it is predicted that future research on hydrogel fire extinguishants can focus on the following aspects: 

In terms of material synthesis and processing, establishing synthetic methodologies and optimizing processes for multifunctional natural fire extinguishing hydrogel. In the in-depth study of the application of hydrogel fire extinguishants, we have observed their ability to form a stable gel film on the surface of combustible materials, which necessitates hydrogels possessing excellent mechanical strength and adhesion properties. To further optimize this performance, it is necessary to explore cutting-edge processing technologies that can enhance both the mechanical strength and adhesion properties of the hydrogels simultaneously [103,104]. Further developing hydrogel foaming technology and establishing efficient hydrogel foaming processes. However, in the research of hydrogel foam fire extinguishants, special attention should be paid to their biosafety. For inevitable pollutants, sufficient consideration should be given to water purification and pollutant degradation issues [105,106,107].

In terms of performance evaluation, systematically studying the fire extinguishing performance, thermal insulation, adhesiveness, rheological properties, and other characteristics of new natural polymer hydrogel fire extinguishing materials with different structures and morphologies. Conducting fundamental research on applications such as spray dynamics and mathematical modeling. Providing theoretical references and material selection for the design and product development of clean and efficient hydrogel fire extinguishing materials.

In terms of product development and application, based on natural polymer hydrogel fire extinguishing materials, a series of new products such as hydrogel additives for firefighting vehicles, household mini hydrogel fire extinguishers, specialized hydrogel fire extinguishing guns, and hydrogel thermal insulation and fire extinguishing mats can be developed. In the future, hydrogels hold tremendous potential in fire extinguishing and prevention. The integration of hydrogel fire extinguishing agents with color-changing intelligence [108] and sensing intelligence [109,110] will greatly promote the application of hydrogels in the field of firefighting, and the development of fully automated integrated hydrogel fire extinguishing and prevention systems is anticipated.

## Figures and Tables

**Table 1:** Different types of hydrogel extinguishants.

Types of Hydrogel Extinguishants	Foam Type	Dry Water Type	Thermosensitive Type	Common Type
The key components	Surfactants	SiO_2_	N-isopropylacrylamide	Natural polymers
Characteristics	Suitable for oil fire	Physical and chemical dual inhibition	Smart stive-responsive	High adhesion
Forms	Foam	Powder	Solution	Sol

**Table 2:** Thermosensitive hydrogel extinguishants.

Gelling Agents	Crosslinking Agents	Initiators	Crosslinking Ways	References
MC, PEG	MBA	KPS	Free-radical copolymerization	[63]
Xylan, CMC, NIPAM, AMPS	MBA	APS	Free-radical copolymerization	[64]
AMPS, pretreated straw	MBA	KPS	Free-radical copolymerization	[65]
MC, PAAS	MgCl_2_	-	Ionic crosslinking	[66]
NIPAM, SA	MgCl_2_	-	Ionic crosslinking	[67]

**Table 3:** Hydrogel foam extinguishants.

Foaming Agents	Gelling Agents	Crosslinking Agents	References
AES, HP	SA	CaCl_2_	[68]
AES, APG, SDS, CTAB	MPS, CMC, PEG-1500	-	[69]
SDBS, SDS, SLS, AES	SA	CaCl_2_	[70]
AOS, AES	CMC	PAC	[71]
APG	XG	PAC	[72]

**Table 4:** Hydrogel dry water extinguishants.

Main Component	Gelling Agents	M_SiO_2__:M_H_2_O_	References
SiO_2_, H_2_O	Carrageenan	0.9:10	[73]
SiO_2_, H_2_O	SA, CS	0.9:10	[74]
SiO_2_, H_2_O	Lignin-based hydrogel, NIPAm	1:9	[75]
SiO_2_, H_2_O	Gellan	5:95	[76]

## Data Availability

The data supporting this study’s findings are available from the corresponding author upon request.
